# COVID-19 Infection and Vaccination and Its Relation to Amyloidosis: What Do We Know Currently?

**DOI:** 10.3390/vaccines11071139

**Published:** 2023-06-24

**Authors:** Wing-Yin Leung, Henry H. L. Wu, Lauren Floyd, Arvind Ponnusamy, Rajkumar Chinnadurai

**Affiliations:** 1 Department of Renal Medicine, Lancashire Teaching Hospitals NHS Foundation Trust, Preston PR2 9HT, UK; wingyin.leung@lthtr.nhs.uk (W.-Y.L.); lauren.floyd@lthtr.nhs.uk (L.F.); arvind.ponnusamy@lthtr.nhs.uk (A.P.); 2 Renal Research Laboratory, Kolling Institute of Medical Research, Royal North Shore Hospital & The University of Sydney, Sydney, NSW 2065, Australia; honlinhenry.wu@health.nsw.gov.au; 3 Faculty of Biology, Medicine and Health, University of Manchester, Manchester M13 9PG, UK; 4 Department of Renal Medicine, Salford Royal Hospital, Northern Care Alliance Foundation Trust, Salford M6 8HD, UK

**Keywords:** COVID-19, amyloidosis, COVID-19 vaccination, outcomes, pathophysiology

## Abstract

Amyloidosis is a complex disorder characterized by deposited insoluble fibrillar proteins which misfold into β-pleated sheets. The pathogenesis of amyloidosis can vary but can be the result of immune dysregulation that occurs from sustained high inflammatory states, often known as AA amyloidosis. Multi-organ involvement including hepatic, gastrointestinal, renal, cardiac and immunological pathological manifestations has been observed amongst individuals presenting with amyloidosis. The recent global pandemic of severe acute respiratory syndrome coronavirus 2, also referred to as coronavirus 2019 (COVID-19), has been shown to be associated with multiple health complications, many of which are similar to those seen in amyloidosis. Though COVID-19 is recognized primarily as a respiratory disease, it has since been found to have a range of extra-pulmonary manifestations, many of which are observed in patients with amyloidosis. These include features of oxidative stress, chronic inflammation and thrombotic risks. It is well known that viral illnesses have been associated with the triggering of autoimmune conditions of which amyloidosis is no different. Over the recent months, reports of new-onset and relapsed disease following COVID-19 infection and vaccination have been published. Despite this, the exact pathophysiological associations of COVID-19 and amyloidosis remain unclear. We present a scoping review based on our systematic search of available evidence relating to amyloidosis, COVID-19 infection and COVID-19 vaccination, evaluating current perspectives and providing insight into knowledge gaps that still needs to be addressed going forward.

## 1. Introduction

Amyloidosis is an umbrella term for a group of rare disorders caused by extracellular deposition of amyloid fibrils. Amyloid fibrils are misfolded and insoluble proteins which subsequently lead to organ dysfunction and death [1,2]. The clinical manifestation of amyloidosis varies but primarily depends on the precursor protein and the organs involved [1,2]. Systemic amyloidosis can be classified by the type of amyloid protein produced [3]. Commonly affected organs include the heart, kidneys, liver, lungs and the gastrointestinal and nervous systems [4]. Systemic light-chain (AL) amyloidosis remains the most common type, although there has been an increase in cases of wide-type transthyretin-associated (ATTRwt) amyloidosis in the past decade [5]. AA amyloidosis is another type of systemic amyloidosis which occurs as a result of abnormal aggregations of serum amyloid A protein, typically caused by periods of prolonged infection, chronic illness or high inflammatory states [6]. With this in mind, the impact of coronavirus 2019 (COVID-19) on the pathogenesis, management and treatment of amyloidosis has been increasingly considered.

Since the World Health Organization (WHO) formally declared the COVID-19 pandemic on 11 March 2020 worldwide healthcare systems have had to adapt their service deliveries to cope with the effects of the pandemic [7]. This includes clinical services involved in the ongoing management of patients with chronic diseases, such as amyloidosis. As of 12 April 2023, there have been 762,791,152 confirmed cases of COVID-19, including 6897,025 deaths, reported to WHO around the world [8]. The introduction of COVID-19 vaccination programs has become an integral part of the public health strategy to reduce cases of COVID-19 worldwide. Over 13 billion vaccine doses have been administered globally as of 9 April 2023 [8].

Whilst COVID-19 infection was initially considered to be primarily affecting the respiratory system, subsequent evidence has shown that it is in fact a complex disorder affecting multiple body systems, in much the same way amyloidosis does [9,10,11,12,13]. With multiple case reports and series reporting new and relapsing amyloidosis following COVID-19 infection and vaccination, the pathogenic link between the two conditions remains a novel area of interest. Given the complexity and multi-dimensional etiologies of amyloidosis, it is expected that COVID-19 infection and vaccination will have different pathogenic effects for each amyloid disease subtype. Research into the cytotoxic effects of COVID-19 and the subsequent production of abnormal proteins is an ongoing research area of significant clinical interest [14]. In this paper, we present a scoping review of the existing literature relating to amyloidosis, COVID-19 infection and vaccination.

## 2. Search Process for Scoping Review

### 2.1. Eligibility Criteria

All full-text research articles reporting amyloidosis in adult patients (age ≥18 years) with COVID-19 infection and/or COVID-19 vaccination were included for consideration following our initial systematic search. Only full-text articles published in the English language were eventually selected. Articles published between 1 December 2019 and 10 April 2023 were included for evaluation in our scoping review. Full-text articles that involved human subjects age <18 years, not published in English language, with a lack of clarity regarding a prospective diagnosis of amyloidosis (i.e., including scenarios where only post-mortem diagnoses were made), published prior to December 2019, or that included non-human subjects were excluded from our detailed, qualitative evaluation.

### 2.2. Search Strategy and Study Selection

**Figure 1:**
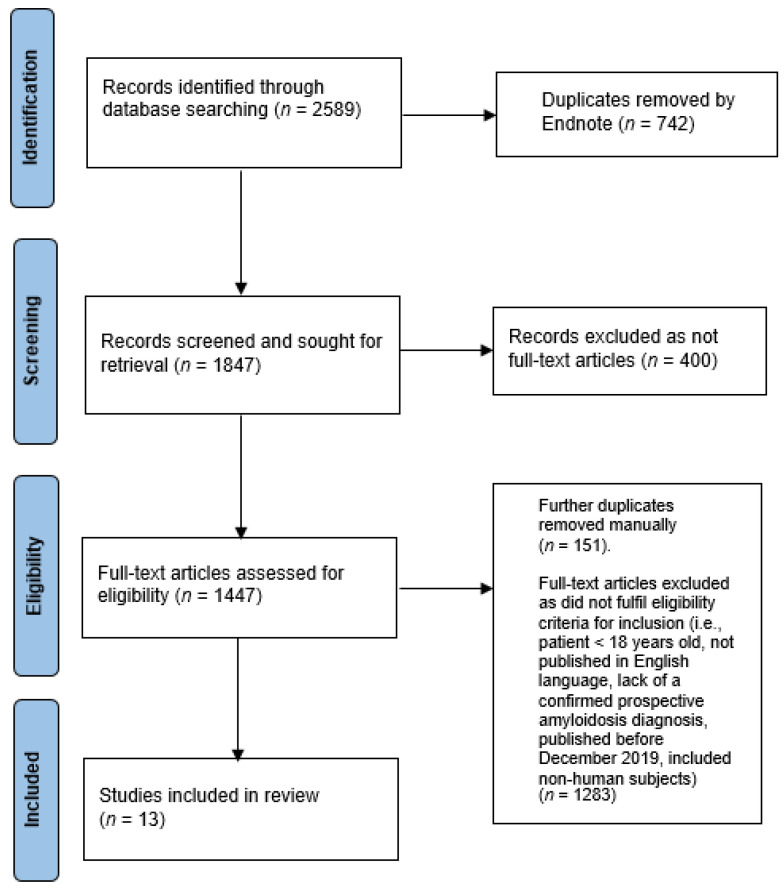
PRISMA Flow Diagram.

A systematic literature search was conducted by two independent authors (W.-Y.L. and H.H.L.W.) in the following databases: ‘PubMed’, ‘Web of Science’, ‘EMBASE’, ‘Medline-ProQuest’. The search terms incorporated the following: (((((((COVID) OR (COVID-19)) OR (Coronavirus)) OR (SARS-CoV-2)) OR (COVID-19 Vaccination)) OR (SARS-CoV−2 Vaccination))) AND ((((Amyloidosis) OR (Amyloidoses)) OR (Amyloid)) OR (Amyloid formation)). The articles were screened by W.-Y.L. and H.H.L.W. for relevance and duplicate publications were removed. Duplicate screening and the eligibility check was performed by both W.-Y.L. and H.H.L.W. The preferred reporting items for systematic reviews and meta-analyses (PRISMA) guidelines were followed to identify, screen and include relevant articles (Figure 1).

### 2.3. Summary of Search Outcome

A total of 2589 articles were identified from our initial search. After excluding duplicates, non-full text articles and full-text articles which did not fulfill the inclusion criteria or were part of the exclusion criteria (amounting to 2576 items), 13 articles were selected for a detailed, qualitative evaluation.

## 3. Clinical Outcomes of COVID-19 Infection in Patients with Amyloidosis

COVID-19 was of grave concern for all individuals with underlying health problems and chronic diseases. During the course of the pandemic, emerging evidence showed that patients with existing and underlying health conditions were at higher risk of severe infection and adverse clinical outcomes, including death. Our search process retrieved three articles reporting clinical outcomes in patients with amyloidosis diagnosed with COVID-19 infection, defined by the detection of positive COVID-19 infection following polymerase chain reaction (PCR) testing (Table 1).

The American Society of Hematology Research Collaborative COVID-19 Registry study [15] evaluated outcomes of 250 patients with hematologic malignancies diagnosed with COVID-19 infection. Amongst the 250 patients, 16% of them either had multiple myeloma (MM) or AL amyloidosis. Within this subgroup, 81% of the MM or AL amyloidosis patients had moderate or severe COVID-19 infection. Moderate infection was defined as needing general hospital admission and severe infection was defined as requiring intensive care unit admission. Whilst the specific breakdown of the amyloidosis group was not reported, these patients who were admitted to hospital had a significantly raised mortality rate of 28%. Similar findings were presented by Lewis et al. [16], who evaluated a cohort of 152 ATTR patients and 103 AL amyloidosis patients during the period between January 2020 and April 2022. They found that patients with amyloidosis were at high risk of severe infection and mortality from COVID-19, in particular those of older age and receiving active immunotherapy. They also found that when comparing the mortality of amyloidosis with pre-pandemic years, there was an excess mortality of 128% in the ATTR cohort and 75% in the AL amyloidosis cohort in 2021, although only one case of mortality was proven to be directly caused by COVID-19.

Ho et al. [17] studied a much larger cohort of 9225 patients with MM or AL amyloidosis. This was a multi-center US study carried out between January 2020 and August 2021. The overall prevalence of COVID-19 infection was approximately 2% during this time period. Within the AL amyloidosis subgroup alone, of 13 patients affected by COVID-19, there were only 4 (30.8%) who required hospitalization, in which 3 (23.1%) of them developed severe symptoms, whereas 1 (7.8%) required intensive care unit admission. It was a positive result that none of the AL amyloidosis patients died during acute admission. As the study covered the time period in which there was rapid development of the COVID-19 vaccination programs globally, Ho et al. reported an increasing rate of patients receiving COVID-19 vaccinations as the study progressed.

Whilst these studies were small, it is clear that COVID-19 had a significant impact on those with amyloidosis. Despite the heterogeneity between the various amyloidosis subtypes, and different time periods during the pandemic in which these studies were conducted, the risk of hospitalization, morbidity and mortality associated with COVID-19 infection increased in comparison to the general population. Age, comorbidities and organ-related dysfunction from amyloid deposits have all been identified as risk factors and the impact of immunosuppressive treatment also played a part in the adverse outcomes [18]. The vaccine uptake in this patient group was good, which likely offered a degree of protection from severe COVID-19 infection and further mortality in the later months of the pandemic. Further registry level studies are needed to determine if there was any significant impact on the new diagnosis of amyloidosis during this time period.

## 4. Impact of COVID-19 Pandemic on the Management of Amyloidosis

The COVID-19 pandemic played a consequential role on various facets of care in patients with amyloidosis: it had an impact on delayed and missed diagnosis of amyloidosis; it reduced and/or delayed immunosuppressive treatment and termination of stem cell transplantation; it impacted vaccination uptake and effectiveness; and it also had a notable impact on obstructions in clinical service delivery.

In the early days of the pandemic, the adaptation strategy to counter COVID-19 revolved around minimizing the transmission of the disease and shielding vulnerable individuals [19]. Since the initial days of the pandemic, it was evident that patients with amyloidosis would represent a particularly vulnerable group. The combination of quarantine measures and the disruption of non-emergency healthcare services resulted in reduced access to healthcare for patients with chronic diseases, such as amyloidosis [20,21]. Furthermore, those with new or relapsing disease were likely to experience delays in diagnostics and treatment facilities were often limited and or restricted [16,18]. Telemedicine has been reported as one of the adaptation measures which enhanced the continuation of care for amyloidosis patients during the early stages of the pandemic [16,18,22,23]. However, the protective role of telemedicine in preventing exposure to COVID-19, might have inevitably come at the cost of sub-optimal care that is delivered virtually. Lewis et al. [16] reported a 36% reduction in the diagnosis of AL amyloidosis in 2020 compared to 2019. This likely reflects a reduction in patients self-presenting due to fear of accessing healthcare services or attributing systemic symptoms as not significant enough to warrant a clinician review. Delays in referral from primary care services and reduced access to investigations as well as the increase in remote reviews via telemedicine may have contributed to delayed or missed diagnosis of amyloidosis cases [16,22].

The pathophysiology of amyloidosis associated with immunoparesis and heightened susceptibility and severity of infection, together with its management involving potent immunosuppressant agents raised great concern, particularly in the initial phases of the pandemic where there was absence of an effective treatment or vaccination [23,24]. Alterations in the management regimen of amyloidosis patients have mainly included modifications to the immunosuppression treatment regimen (either by reducing the dose or the duration of its course), and deferring invasive procedures, such as organ biopsies, autologous hematopoietic stem cell and renal transplantation [22,23,25]. Considering a longer than expected critical period of the pandemic where there were many disruptions in clinical services, such as the delay or termination of autologous stem cell transplantation, a key definitive treatment measure in amyloidosis, significantly impacted the management and clinical outcomes of amyloidosis patients [26].

As the clinical trials for the efficacy of COVID-19 vaccines in healthy individuals showed promising results, the scientific community debated how best to pursue rollout of the COVID-19 vaccines. The post-vaccination phase of the COVID-19 pandemic was characterized by a particular focus on the risk–benefit analysis of vaccination versus infection in different sub-populations, in particular vulnerable individuals. The majority of clinical trials excluded immunosuppressed patients, such as those with amyloidosis, on active chemotherapy and therefore data on the efficacy in such patients were limited [17,26]. Following the introduction of updated recommendations advocating full vaccination and booster doses for vulnerable patients, an increasing number of amyloidosis patients received COVID-19 vaccinations [27].

In regard to vaccine efficacy in the amyloidosis population, previous studies showed variable rates of seroconversion in patients with amyloidosis receiving other vaccines, suggesting this group of patients may be at risk of reduced humoral response to vaccinations [28,29]. Later studies have dismissed these concerns with findings that there are comparable seroconversion rates between patients with monoclonal gammopathies and heathy individuals following COVID-19 vaccination [30,31]. The role of immunosuppression, specifically for patients receiving B cell depleting treatments, such as rituximab, has been well-documented to cause reduced seroconversion for numerous pathologies [32,33]. However, studies which involved patients with amyloidosis and monoclonal gammopathies receiving immunosuppression and COVID-19 vaccination are mostly inconclusive or insufficiently powered to determine whether immunosuppression was associated with a poor antibody response post-vaccination, as well as breakthrough infections [31,34,35].

Considerations were also made to the possible risks induced from COVID-19 vaccination, in particular the thromboembolic risks. Amyloidosis is associated with increased blood viscosity and similar effects were seen post vaccination [14,36]. Whilst there has been no evidence of increased venous thromboembolic incidence in those with amyloid, the monitoring of thrombohemostasis during immunization was considered [37].

Healthcare systems have attempted to restore clinical service delivery to the levels preceding the pandemic as safety and efficacy data for the COVID-19 vaccines become more well-established. Current challenges are mostly not related to the pathophysiological effects of COVID-19 for the infected individual(s), but rather the socio-economic factors have been more apparent, such as disruption in the global supply chain, and healthcare services still not back to running at full capacity due to shortages in the healthcare workforce and political unrest [38,39]. For some patients with amyloidosis, the struggles of having to live with a chronic condition alongside the stresses of living through the COVID-19 pandemic with its psychosocial and financial impact, may have led to depressive symptoms [40,41]. Those with ‘long COVID-19′ diagnoses may have lingering symptoms, and together with amyloidosis amongst other comorbidities, may find it challenging to partake in full-time employment, for example, thus leading to financial difficulties. Finding solutions for many of these issues are likely to be far more complex, as it may take years before we can determine fully how these challenges would have played a role in impacting the outcomes and holistic management of amyloidosis patients.

## 5. Reported Cases of COVID-19 Infection-Induced Amyloidosis

Djafari et al. [42] (see Table 2) reported a case of 68-year-old woman with a ten-day history of shortness of breath and fever who tested positive for COVID-19 infection. She had a background of secondary amyloidosis with kidney involvement due to rheumatoid arthritis. She was on immunosuppressive treatment with methotrexate, prednisolone and Etanercept for ten years. She presented with gross hematuria and her radiological investigations were unremarkable. She had a cystoscopy and biopsy of her bladder mucosa which confirmed the diagnosis of amyloidosis of the bladder. She was treated conservatively with bladder irrigation, platelets and packed cell transfusion but later died due to respiratory failure. The gross hematuria, in this case, was attributed to COVID-19 infection-related hemorrhage.

Mir et al. [43] (see Table 2) reported the case of a 55-year-old gentleman who had COVID-19 infection with lung involvement needing hospitalization. He recovered with supportive management (oxygen, antibiotics, anti-coagulants and steroids) and his creatinine was normal during this admission (0.9 mg/dL). Five months later, he presented with fatigue, malaise and unexplained acute kidney injury (creatinine was 6.04 mg/dL). There was no relevant positive medical history or investigation finding (immunology and virology screen) to explain for the renal dysfunction. His kidney biopsy confirmed the diagnosis of renal AA amyloidosis. He was treated with steroids and colchicine with creatinine showing signs of recovery in four weeks (3.8 mg/dL). A strong temporal association supported the link with COVID-19 infection.

Otherwise, there were numerous reports of cardiac amyloidosis found on autopsy examination in patients who were previously diagnosed with COVID-19 [44,45,46,47]. However, the medical history and conclusions from autopsy examination suggest that these cases of amyloidosis are most likely chronic or senile, preceding that of acute COVID-19 infection. It is difficult to demonstrate causative associations between COVID-19 and amyloidosis manifestations within a post-mortem scenario.

## 6. Proposed Pathophysiological Associations of COVID-19 Infection-Induced Amyloidosis

Emerging reports of new-onset and relapsed amyloidosis manifestations following acute COVID-19 infection have led to debate and postulations on the relationship between COVID-19 and amyloidosis at a pathophysiological level. It has been hypothesized is that the severity of COVID-19, in terms of its pulmonary and systemic inflammatory complications, would most certainly generate increased protein aggregation and amyloid formation.

The serum amyloid A (SAA) has been demonstrated to be a sensitive marker of acute phase response and occur in association of systemic infection, inflammation and cytokine storms [48,49]. It is unsurprising therefore that COVID-19 severity has been linked to high levels of SAA [48,49]. The SAA protein structure exists in various forms. Its hexamer is the biologically active form which carries lipids around the body system during inflammatory states [50]. Its monomers are prone to enzymatic cleavage, forming small fragments which then form amyloid fibrils [50]. SAA levels would increase with the progression of COVID-19 to a thousand-fold more than its baseline level in acute COVID-19 illness [51]. Typically, such abnormally high levels are characteristic in patients with cancer or chronic inflammatory diseases (i.e., rheumatological conditions, such as systemic lupus erythematous), who often have systemic amyloid deposition with its associated damage [52].

Zinellu et al. [48] conducted a meta-analysis of SAA concentrations in COVID-19 patients and the association of disease severity and mortality. When analyzing a total of 5617 patients from 19 studies they showed increasing SAA concentrations correlated with worse COVID-19 infection measured by mortality and acute respiratory distress syndrome (ARDS). This suggests that SAA levels could be used as a way of risk stratifying those vulnerable to worse clinical outcomes. However, what remains to be seen, is the long-term risk this poses to the development of amyloidosis. Some studies showed a quick reduction in SAA levels back to baseline but the risk for those with prolonged infection remains unknown [53]. It is also worth noting that those with pro-inflammatory conditions, such as malignancy, diabetes and autoimmune disease have been shown to have high SAA levels and during the pandemic these patient groups had worse outcomes and more severe disease suggesting a pathological link that remains not fully understood [48].

**Figure 2:**
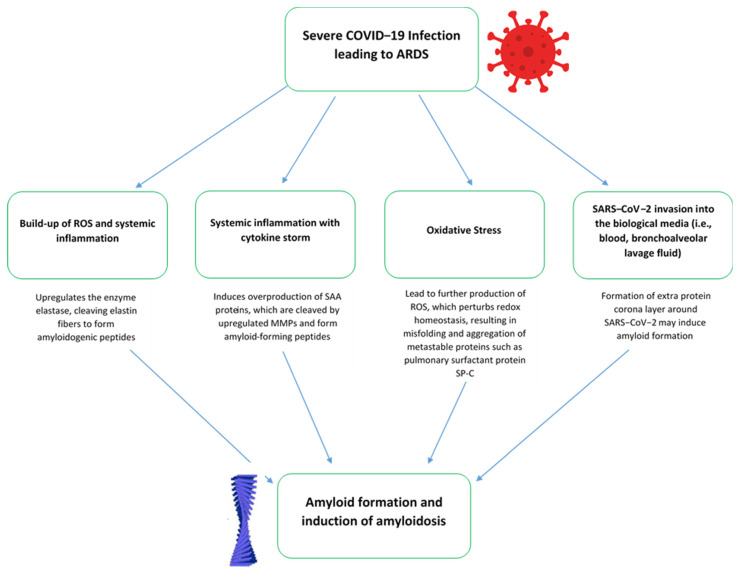
Pathophysiological pathways of COVID-19 infection-induced amyloidosis.

Figure 2 summarizes the pathophysiological pathways of COVID-19 infection-induced amyloidosis based on current understanding. Increases in SAA protein levels during COVID-19 infection may be spurred on either from an initially mild and asymptomatic phase of disease, followed by pulmonary infection and then a systemic inflammatory phase of disease, or in severe cases, drastic increases in SAA protein levels due to ARDS [54]. During the pulmonary phase of COVID-19 infection, entry of SARS-CoV-2 particles inside the pulmonary alveoli and macrophage engulfment of these particles leads to chemokine release [54,55]. Chemokines act as signaling molecules for circulating macrophages and neutrophils which are then activated [56]. Neutrophils upregulate the expression of cytokines and proteases that is responsible for inducing systemic inflammation and injury to epithelial and endothelial cells [57]. Subsequent loss in the permeability of endothelial cells allows fluid to enter the pulmonary alveoli, leading to hypoxia and dyspnea symptoms [54]. Inflammatory responses generated from these processes would then upregulate the enzyme elastase which might cleave elastin fibers to form amyloidogenic peptides [58]. Ongoing inflammation and subsequent cytokine (i.e., interleukin−1 or 6) activation will affect other systemic organs, such as the liver, heart and kidneys in which SAA overproduction could be observed [54]. SAA is cleaved by upregulated matrix metalloproteases and forms amyloid-forming peptides. The introduction of oxidative stress, which is induced from progressing SARS-CoV-2 infection, systemic inflammation and ARDS in severe cases, would lead to reactive oxygen species (ROS) production [59,60]. ROS production stimulates redox homeostasis, which results in the misfolding and aggregation of metastable proteins, such as pulmonary surfactant protein SP-C [54,61]. If SARS-CoV-2 reaches the bronchoalveolar lavage fluid, or invades into the bloodstream, it may form an extra protein corona layer itself which induces further amyloid formation [58].

## 7. Reported Cases of COVID-19 Vaccination Induced Amyloidosis

Shahandeh et al. [62] (see Table 2) reported a 54-year-old woman with a background of recent non-ischemic cardiomyopathy attributed to COVID-19 vaccination-induced myocarditis presenting with cardiogenic shock. She received mechanical circulatory support followed by uncomplicated heart transplantation. The explanted heart pathology confirmed a diagnosis of kappa AL amyloidosis. She was commenced on daratumumab monotherapy with no evidence of disease relapse on surveillance endomyocardial biopsies.

**Table 2:** Summary of prospectively diagnosed cases reported in the literature relating to COVID-19 infection and COVID-19 vaccination-induced amyloidosis.

Author and Year	Age (years)	Sex	New Onset or Relapse	Comorbidities	Primary Management	Organ Involved	Presentation	Biopsy	Treatment Received	Clinical Outcome
COVID-19 Infection										
Djafari et al., (2021) [42]	68	F	Relapse	Rheumatoid arthritis	Methotrexate Prednisolone Etanercept	Urinary Bladder	Gross hematuria	Bladder mucosa: AA Amyloidosis	Conservative management (bladder irrigation, platelet, and packed cell transfusion)	Died due to respiratory failure
Mir et al., (2023) [43]	55	M	New onset	None	-	Kidney	Acute kidney injury (unexplained)	Kidney: Renal AA Amyloidosis	Prednisolone and Colchicine	Recovering following treatment initiation
COVID-19 Vaccination										
Shahandeh et al., (2023) [62]	54	F	New onset	Non-ischemic cardiomyopathy to COVID-19 vaccine associated myocarditis	-	Heart	Cardiogenic shock	Heart: AL Amyloidosis	Heart transplant Daratumumab	Recovered following treatment

There was also one other case of cardiac amyloidosis which appears to have developed following COVID-19 vaccination, though this was diagnosed upon post-mortem examination. Hansen et al. [63] made a histological diagnosis of cardiac amyloidosis in a previously well and symptomless 86-year-old man who died following a single dose of mRNA Pfizer-BioNTech COVID-19 vaccination. Nevertheless, the post-mortem nature of this discovery makes it difficult to ascertain direct correlations between these two events.

Another case of potential COVID-19 vaccination -induced amyloidosis describes a 60-year-old male of Laos descent who has been in Australia since the 1980s presenting with acute onset of peripheral edema and weight gain one week after receiving the second dose of mRNA Pfizer-BioNTech COVID-19 vaccine [64]. This case was not included in the final article list from our search given it was presented as a conference abstract at the 2022 Australia and New Zealand Society of Nephrology Meeting. The patient had no past medical history and was not taking regular medications, including over the counter remedies. Subsequent renal biopsy showed Congo red staining of amyloid deposits, and the bone marrow biopsy demonstrated 10% plasma cell aggregates via CD138 and amyloid deposit within the extramedullary tissue. At the time of this abstract’s publication, the patient had received bortezomib, cyclophosphamide and dexamethasone chemotherapy without hematological response and was awaiting work-up for a stem cell transplant.

## 8. Proposed Pathophysiological Associations of COVID-19 Vaccination-Induced Amyloidosis

The paucity of reported cases describing COVID-19 vaccination-induced amyloidosis makes it challenging to decipher the pathophysiological associations which may explain this presentation. Nonetheless, raised SAA levels have been observed in other pathologies where there is COVID-19 vaccine-induced disease. One frequently discussed example is in COVID-19 vaccine-induced IgA disease (vasculitis and/or nephropathy), where marked SAA level increases were observed [65,66]. There are also other autoimmune conditions where SAA level elevations were noted [67]. The molecular mechanisms of how COVID-19 vaccination induces these disease manifestations remain largely unestablished. It has been postulated that the occurrence of these adverse effects appear as the result of acute inflammation caused by both the SARS-CoV-2 virus and the vaccine, given the symptomatic complications for many of these autoimmune conditions are similar in both viral-induced and vaccine-induced states [68,69]. The SARS-CoV-2 S protein, a common denominator between the virus and the vaccine, and soluble or endothelial cell membrane-attached angiotensin-converting enzyme 2 (ACE-2) is thought to play a significant role in the pathophysiological process, particularly if it enters the circulation and is systematically distributed around the human body [68,70,71]. More novel basic scientific evidence have suggested the potential of ionizable lipids within S protein mRNA-containing lipid nanoparticles to trigger pro-inflammatory responses following mRNA vaccination, by the activation of Toll-like receptors [72,73]. It has been found that lipid nanoparticles used in nucleoside-modified mRNA vaccine mice studies are highly inflammatory, evidenced by excessive neutrophil infiltration, activation of diverse inflammatory pathways, and production of various cytokines and chemokines [73]. This may have subsequently stimulated the processes of amyloid formation as described in the ‘proposed pathophysiological associations of COVID-19 induced amyloidosis’ section. Inflammatory responses via the lipid nanoparticles pathway are thought to be more severe in those with pre-existing inflammatory or autoimmune disease (e.g., if they have pre-existing amyloidosis) as demonstrated in a mouse model administration of mRNA–lipid nanoparticles, wherein this effect was shown to be specific to the lipid nanoparticle pathway only [74].

## 9. Conclusions

This scoping review provides an updated summary into the potential associations between amyloidosis, COVID-19 infection and COVID-19 vaccination. The challenge of maintaining ideal standards of management for patients with chronic amyloidosis during the pandemic is understated. Whilst global vaccination programs have been integral to the protection of patients with amyloidosis and others with chronic diseases, ensuring adequate responses to vaccination in this complex group of patients remains an important issue to address.

Many amyloidosis patients concurrently receive immunosuppressive treatment and vaccination(s), which may affect humoral response and seroconversion to vaccinations. It is a conundrum on how we could restore service delivery for the amyloidosis population back to prior levels in optimizing patient outcomes as much as possible. With virtual healthcare now being part of a new landscape of patient care, early identification of the pathophysiological and histopathological processes relating to new-onset amyloid formation and relapsing amyloidosis following COVID-19 infection and vaccination would also be important, with timely intervention most likely to improve patient outcomes. In relation to COVID-19 vaccination-induced cases, it should be stressed that the presently reported cases are novel and isolated in relation to the hundreds of millions of vaccinations that have occurred, and the protective benefits offered by COVID-19 vaccination far outweigh its risks, particularly for those living with chronic comorbidities. Research is needed to clarify the pattern of these links, if any. The creation of national and international study groups aiding systematic collection of observational data to determine associations between COVID-19 infection, COVID-19 vaccination and amyloidosis would be helpful. This may allow further conclusions to be drawn in relation to the similarities and differences of these associations, across varying patient demographics and disease subgroups. Establishing clinical trials to investigate management pathways and novel treatment options for this scenario is also anticipated (e.g., CAR-T cell therapy, which is increasingly used outside oncology for autoimmune disease and viral infections, may be an option within this context) to optimize outcomes for patients [75].

Overall, we are encouraged by the growing scientific interest in this topical area, as progression with research initiatives will hopefully provide further answers and solutions to the current unknown factors that hinder our management of this complex patient population.

## Figures and Tables

**Table 1:** Literature reporting health outcomes for COVID-19 infection in patients with amyloidosis.

Author, Year	Study Period	Cohort and Subgroups	Location of Study	Vaccination Rate	COVID-19 Infection Rate	Clinical Outcomes
Wood et al., 2020 [15]	April–July 2020	Hematological malignancy with COVID-19 250 pts), of which 40 pts (16%) had MM or AL	Worldwide (65% in North America)	N/A	N/A	30/37 (81.1%) with moderate to severe infection, 11/39 (28% mortality)
Lewis et al., 2022 [16]	January 2020–April 2022	ATTR (152 pts) and AL (103 pts)	Alberta, Canada	ATTR 137/152 (90.6%) vaccinated. AL 84/103 (81.6%) vaccinated.	ATTR 78/131 (59.5%) tested 4/78 (5.1%) PCR +ve AL 42/60 (70%) tested 11/42 (26.2%) PCR +ve 6/15 (40%) PCR +ve patients were unvaccinated	4 patients required hospital admission (2 AL patients both vaccinated; 2 ATTR patients both unvaccinated) 1 death caused directly by COVID-19 infection (ATTR patient, not vaccinated)
Ho et al., 2023 [17]	January 2020–August 2021	MM and AL (9225 pts)	United States	187/9225 (2%) (174 MM.; 13AL)	187/9225 (2%) (174 MM.; 13 AL)	3/13 (23.1%) with severe infection, 4/13 (30.8%) required hospital admission, 1/13 (7.8%) required ICU admission, 9 patient deaths (9 MM.; 0 AL) 2 patients (17%) with severe infection; 0 deaths

## Data Availability

No new data were created in this manuscript.

## Abbreviations

AL: Light Chain Amyloidosis; ARDS: Acute Respiratory Distress Syndrome; ATTR: Transthyretin Amyloidosis; COVID-19: Coronavirus 2019; F: Female; ICU: Intensive Care Unit; IDD: Immunoglobulin Deposition Disease; M: Male; MM: Multiple Myeloma; MMP: Matrix Metalloproteases; PCR +ve: Polymerase Chain Reaction Test Positive; ROS: Reactive Oxygen Species; SAA: Serum Amyloid A.; SP-C: Surfactant Protein C
